# Interobserver Agreement in Magnetic Resonance of the Sacroiliac Joints in Patients with Spondyloarthritis

**DOI:** 10.1155/2017/3143069

**Published:** 2017-02-13

**Authors:** Juan C. Rueda, Sofia Arias-Correal, Andres Y. Vasquez, Enrique Calvo, Paola Peña, Marlon Porras, Jose-Ignacio Angarita, Eugenia-Lucia Saldarriaga, Ana M. Santos, John Londono

**Affiliations:** ^1^Department of Rheumatology, Universidad de La Sabana-Hospital Militar Central, Chía, Colombia; ^2^Department of Radiology, Universidad Nacional de Colombia, Bogotá, Colombia

## Abstract

*Background*. Clinical, laboratory, and radiologic parameters are used for diagnosis and classification of spondyloarthritis (SpA). Magnetic resonance imaging (MRI) of sacroiliac (SI) joints is being increasingly used to detect early sacroiliitis. We decided to evaluate the interobserver agreement in MRI findings of SI joints of SpA patients between a local radiologist, a rheumatologist, and an expert radiologist in musculoskeletal diseases.* Methods*. 66 MRI images of the SI joints of patients with established diagnosis of SpA were evaluated. Agreement was expressed in Cohen's kappa.* Results*. Interobserver agreement between a local radiologist and an expert radiologist was fair (*κ* = 0.37). Only acute findings showed a moderate agreement (*κ* = 0.45), while chronic findings revealed 76.5% of disagreement (*κ* = 0.31). A fair agreement was observed in acute findings (*κ* = 0.38) as well as chronic findings (*κ* = 0.38) between a local radiologist and a rheumatologist. There was a substantial agreement between an expert radiologist and a rheumatologist (*κ* = 0.73). In acute findings, a 100% agreement was achieved. Also chronic and acute plus chronic findings showed high levels of agreement (*κ* = 0.73 and 0.62, resp.).* Conclusions*. Our study shows that rheumatologists may have similar MRI interpretations of SI joints in SpA patients as an expert radiologist.

## 1. Background

The spondyloarthritis (SpA) is a group of interconnected inflammatory arthritides that share multiple clinical features and common genetic factors [1]. Men with a clinical onset before 50 years are more frequently affected [2, 3]. The main clinical manifestations are inflammatory back pain, peripheral arthritis, enthesitis, and uveitis, while other organ manifestations are rare [4].

Clinical, laboratory, and radiologic parameters are used for its diagnosis and classification. During the last 30 years, multiple classification criteria have been proposed; however one of the most accepted and used is the European Spondyloarthropathy Study Group (ESSG). The ESSG categorizes SpA in Ankylosing Spondylitis (AS), Psoriatic Arthritis (PsA), Reactive Arthritis (ReA), Arthritis associated with Inflammatory Bowel Disease (AIBD), and Undifferentiated Spondyloarthritis (uSpA) [5].

Recently, the Assessment of SpondyloArthritis International Society (ASAS) developed new classification criteria for the early recognition of SpA, which differentiates two predominant manifestations of SpA: axial (axSpA) and peripheral (pSpA) disease [6, 7]. According to ASAS, axSpA is defined as the presence of sacroiliitis by radiography or magnetic resonance imaging (MRI) plus at least one SpA feature (imaging arm) or the presence of HLA-B27 plus at least two SpA features (clinical arm) [6].

However, the recognition of sacroiliitis on conventional radiographs can be challenging because of the anatomic complexity of the sacroiliac (SI) joints, which leads to misinterpretations [8]. This is demonstrated in previous studies where interobserver (*κ* = 0.19 to 0.79) and intraobserver (*κ* = 0.07 to 1.0) variations differ widely [9–13]. Even more so, a study by van Tubergen demonstrated that rheumatologist as well as radiologist showed modest sensitivity and specificity for sacroiliitis on conventional radiographs, which did not improve significantly with neither individual training nor workshops [14].

Different outcomes are demonstrated in similar studies with the use of MRI. Acute and chronic findings in SI joints showed improvement in interobserver variations (*κ* = 0.38 to 0.80) [13, 15–17]. Also, when comparing conventional radiographs and MRI for detection of chronic structural changes in SI joints, MRI showed better sensitivity and specificity (84% and 61%, resp.) with low agreement concerning definite erosions (*κ* = 0.11), moderate agreement for definite subchondral sclerosis (*κ* = 0.46) and definite joint space abnormalities (*κ* = 0.41), and almost perfect agreement for joint ankylosis (*κ* = 0.85) [13]. Also, even better interobserver agreements for SI erosion on MRI have been reported by Weber et al. with kappa values above 0.70 [15].

Initial diagnostic approach is made by primary care physicians, family physicians, or internal medicine specialists, which relied on an accurate reading of SI images. In daily practice, readings of the SI images are made by local radiologists. However, radiographic interpretations tend to differ according to expertise, as it is demonstrated in a study by Geijer et al. where the presence of sacroiliitis in computed tomography (CT) showed a good interobserver agreement between two expert radiologists (*κ* = 0.67), but it decreased when comparing an expert radiologist reading and a local radiologist reading (*κ* = 0.46) [18]. Therefore, the aim of the present study is to establish the degree of interobserver variation of the MRI readings of acute and chronic changes of SI joints in patients with SpA between local radiologists, an expert musculoskeletal radiologist, and a rheumatologist.

## 2. Methods

### 2.1. Patients

In total, 66 MRI images of the SI joints of patients with established diagnosis of SpA according to the ESSG criteria [5], who attended spondyloarthritis ambulatory clinic of the Hospital Militar Central, between January and December of 2015, were evaluated. All patients were assessed under a previously validated structured protocol. Also, patients were characterized according to the ASAS criteria for further analysis [6, 7]. Exclusion criteria included subjects under 18 years and individuals with malignancies or other rheumatic diseases.

The study was approved by the Ethics Committee of the Hospital Militar Central. All studied subjects provided written informed consent and confidentiality was strictly maintained. The study followed norms established by the Helsinki Declaration, The Guidelines for Good Clinical Practice, and Resolution 8430 (1993) of the Colombian Ministry of Social Protection.

### 2.2. Assessment of Clinical Rheumatologic Parameters

Disease activity was assessed by Bath Ankylosing Spondylitis Disease Activity Index (BASDAI) [19], erythrocyte sedimentation rate (ESR), and high sensitivity C-reactive protein (hsCRP). Functionality was assessed by Bath Ankylosing Spondylitis Functionality Index (BASFI) [20].

### 2.3. Evaluation of MRI

Semicoronal and axial MR slices of the SI joints with T1, T2 sequence, fat suppression technique, and Short Tau Inversion Recovery (STIR) with axial T1 and T2 protocol were taken for each patient in the radiology department of the Hospital Militar Central. The same General Electric MR450 1.5 tesla equipment was used in all patients. All images were read by four blinded local radiologists in charge of all readings of the Hospital Militar Central, without the use of any reading protocol. The readings by the local radiologist included the following findings: normal (no findings), acute (bone marrow oedema), chronic (sclerosis, erosions, fat infiltration, ankylosis, and bony formations), and acute plus chronic (acute plus any chronic finding), which were assessed in a dichotomous way. An expert radiologist in musculoskeletal disease (EC) and a rheumatologist (JL) also read the images blinded to the diagnosis and the local radiologists readings, using the previous mentioned categories (normal, acute, chronic, and acute plus chronic) in order to make comparisons.

Local radiologists are graduated radiologists with no formal experience in musculoskeletal radiology. The expert radiologist (EC) is a radiologist with 30-year experience in musculoskeletal radiology with exclusive dedication to interpreting images in rheumatology, specially in SpA. The rheumatologist (JL) is a professor and coordinator of an outpatient clinic of SpA in a University Hospital (Hospital Militar Central). Both EC and JL have attended and fulfilled several EULAR and ASAS courses on SpA imaging.

### 2.4. Statistical Analysis

Results of the categorical variables were expressed in contingency and frequency tables. For numerical variables measures of central tendency and dispersion that included mean, standard deviation (SD), median, and interquartile range were used.

Agreement was calculated using cross-tabulation expressed in Cohen's kappa [21, 22] for the following comparisons: acute findings between local radiologist, expert radiologist, and a rheumatologist; chronic findings between local radiologist, expert radiologist, and a rheumatologist; and acute plus chronic findings between local radiologist, expert radiologist, and a rheumatologist.

All kappa values were interpreted according to the standards proposed by Landis and Koch, as follows: values 0–0.20 slight, 0.21–0.40 fair, 0.41–0.60 moderate, 0.61–0.80 substantial, and 0.81–1.0 indicating almost perfect agreement [23]. Values between minus 1 and 0 the same categories apply as above 0, but for disagreement. Also percentage of agreement was calculated.

SPSS software version 19.0 for Windows was used for the statistical analysis.

## 3. Results

### 3.1. Characteristics of SpA Patients

The mean age of the 66 patients included in the study was 34.35 ± 11.76 years. Most patients were male (*n*: 41; 62.7%). ReA was the most frequent diagnosis (*n*: 35; 52.5%) and only 13 patients (25.6%) completed the New York criteria.  Table 1 shows the general characteristics of the studied population.

### 3.2. Interobserver Agreement between Local Radiologist and Expert Radiologist

In general, interobserver agreement between a local radiologist and an expert radiologist was fair (*κ* = 0.37). Only acute findings showed a moderate agreement (*κ* = 0.45), while chronic findings revealed high levels of disagreement (*κ* = 0.31) (Table 2).

### 3.3. Interobserver Agreement between Local Radiologist and Rheumatologist

A fair agreement was observed in acute findings (*κ* = 0.38; *p* = 0.071) as well as chronic findings (*κ* = 0.38; *p* = 0.071) between local radiologist and a rheumatologist. An even worse agreement was found in acute plus chronic findings with a *κ* value of 0.19 indicating a slight agreement (*p* = 0.502).

### 3.4. Interobserver Agreement between Expert Radiologist and Rheumatologist

There was a substantial agreement between an expert radiologist and a rheumatologist (*κ* = 0.73). In acute findings, a *κ* value of 0.69 was achieved. Also chronic and acute plus chronic findings showed high levels of agreement (*κ* = 0.73 and 0.62, resp.) (Table 3).

## 4. Discussion

To our knowledge this is the first study to evaluate variations in MRI of SI joints in SpA patients between local radiologists, an expert radiologist, and a rheumatologist. In our study, local radiologists are in charge of interpreting every day studies. This includes plain radiographs, MRIs, CTs, and ultrasonography. Their knowledge in musculoskeletal imaging is limited to what was learned during their radiology training. Some will have more experience than others; however none have specific preparation in musculoskeletal imaging. This could be the reason why we found a fair interobserver agreement of imaging findings between local radiologist and an expert radiologist in musculoskeletal diseases.

A similar study found slightly higher interobserver agreements. In the mentioned study, the original reports of SI joints evaluated with CT were compared to readings from two other expert observers. A moderate agreement was found between the original reports and the readings from the two observers (*κ*=0.46 and *κ* = 0.44) [18].

Other studies have compared readings of radiologists and rheumatologist with different levels of expertise. Also, higher interobserver agreements were found. Van Den Berg et al. found a moderate agreement (*κ* = 0.55) on readings of pelvic radiographs of SI joints of patients with suspicion of SpA between rheumatologists or radiologists and experts [12]. A study with SI joint MRIs found an even higher interobserver agreement (*κ* = 0.70) between readings of local radiologists and rheumatologists and two experts [16]. However, these studies compared the readings of experts and “nonexperts” in musculoskeletal imaging with the knowledge of being in a study when the readings were made.

The expertise in reading MRIs of SI in SpA patients and higher levels of agreement is also corroborated in a study by Arnbak et al. where intra- and interobserver agreement between experts was substantial to almost perfect (*κ* = 0.61 and *κ* = 0.79, resp.) [24]. These findings suggest that the expertise, understood as specific training in musculoskeletal imaging, increases the correct interpretation with almost perfect reproducibility. However, a study in detection of sacroiliitis in plain radiographs of SI joints demonstrated that no improvement in performance was found after individual training sessions and workshops [14]. They explained that possibly the participants after training sessions changed the attitude towards interpreting the radiographs.

This change of attitude towards interpretation in studies can be explained by the Hawthorne effect. Participants tend to be more careful and concerned with accuracy and exactness, a phenomenon of altered behavior resulting from the awareness of being part of an experimental study [25, 26]. This could explain our lower interobserver agreement between experts and “nonexperts.”

Interestingly, our study showed a much better performance of a rheumatologist in the interpretation of MRI of the SI joints when compared to an expert radiologist, achieving substantial agreement throughout the whole gamut of findings. Agreement between an expert radiologist and an expert rheumatologist trained by specialized courses is high and similar for both acute and chronic lesions. There was no relevant difference in agreement for acute (*κ* = 0.69) and chronic lesions (*κ* = 0.73). However, when comparing between local radiologist and a rheumatologist the interobserver agreements were fair at best. Although the Hawthorne effect can explain these results, we also consider that the improved interpretation of SI joints by a rheumatologist is accomplished by specific training during fellowship and constant readings and exposure to musculoskeletal imaging.

It is important to emphasize that the broad community of radiologists (and possibly also many rheumatologists) would need specific training in assessment of axial SpA, as it has become so important for diagnosis/classification and treatment decisions. In most countries, both specialities have no such education in their professional curriculum.

Our study has limitations. First, no specific reading protocol of MRI was used. This can lead to interpretation bias and lack of standardization. Second, two different timelines for data collection were compared. The MRIs interpretations from local radiologists were retrospectively collected whereas rheumatologist and expert radiologist interpretations were prospectively collected. This could increase the Hawthorne effect because local radiologists were unaware that their interpretations were to be used in a study, while the rheumatologist and expert radiologist were aware. Third, a bigger sample size would increase the statistical power.

Finally, more studies are required with strict methodological rigor as well as more sample size to confirm our findings. Also, a study similar to the one by van Tubergen should be performed using MRIs in order to confirm whether proper training improves or not interpretation.

## 5. Conclusions

Our study shows that rheumatologists may have similar MRI interpretations of SI joints in SpA patients as an expert radiologist. We believe that in some degree these results may be altered by the Hawthorne effect. However, it is clear that expertise achieved during rheumatology training as well as constant reviewing of musculoskeletal imaging improves interpretation, which can be reflected in daily rheumatology practice.

## Figures and Tables

**Table 1:** Characteristics of the study population.

	ESSG			ASAS	
	AS	ReA	uSpA	axSpA	pSpA
	(*n*: 20; 30.5%)	(*n*: 35; 52.5%)	(*n*: 11; 16.9%)	(*n*: 50; 76.3%)	(*n*: 55; 83.1%)
Age of onset, years (mean; SD)	25.2 ± 8.6	27.9 ± 6.8	31.4 ± 10.3	31.45 ± 11.0	38.7 ± 10.1
Sex (%)					
Male	12 (60.0)	28 (80.0)	5 (44.0)	24 (47.6)	26 (46.9)
Female	8 (40.0)	7 (20.0)	6 (56.0)	26 (52.4)	29 (53.1)
HLA-B27 (%)	16 (82.3)	21 (61.5)	5 (48.9)	38 (75.2)	36 (65.3)
Symptom duration (median; interquartile range)	6.2 (1.4–12.1)	1.17 (0.2–4.9)	3.0 (1.4–10.1)	3.79 (1–11.6)	3.0 (1–10.7)
ESR (mean; SD)	21.8 ± 17.5	25.6 ± 22.5	16.9 ± 14.8	22.1 ± 19.2	21.4 ± 18.0
hsCRP (median; interquartile range)	0.54 (0.2–4.1)	2.48 (0.2–15.9)	0.38 (0.1–0.5)	0.50 (0.1–2.7)	0.50 (0.2–2.4)
BASDAI (mean; SD)	5.3 ± 2.6	5.1 ± 2.9	5.8 ± 2.2	5.5 ± 2.5	5.66 ± 2.4
BASFI (mean; SD)	5.1 ± 2.5	4.9 ± 2.9	5.1 ± 2.3	5.1 ± 2.4	5.1 ± 2.4

ESSG: European Spondyloarthropathy Study Group; ASAS: Assessment of SpondyloArthritis International Society; AS: Ankylosing Spondylitis; ReA: Reactive Arthritis; uSpA: Undifferentiated Spondyloarthritis; axSpA: axial spondyloarthritis; pSpA: peripheral spondyloarthritis; SD: standard deviation; ESR: erythrocyte sedimentation rate; hsCRP: high sensitivity C-reactive protein; BASDAI: Bath Ankylosing Spondylitis Disease Activity Index; BASFI: Bath Ankylosing Spondylitis Functional Index.

**Table 2:** Interobserver agreement between local radiologist and expert radiologist.

Expert radiologist	Local radiologist				
	Agreement	Disagreement	Total	*κ*	*p*
	*n* (%)	*n* (%)			
Normal	24 (96)	1 (4)	25/66	0.39	0.000
Acute	3 (50)	3 (50)	6/66	0.45	0.000
Chronic	4 (23.5)	13 (76.5)	17/66	0.31	0.000
Acute plus chronic	7 (38.9)	11 (61.1)	18/66	0.34	0.003
Total	38 (57.7)	28 (43.3)	66	0.37	0.000

**Table 3:** Interobserver agreement between expert radiologist and rheumatologist.

Rheumatologist	Expert radiologist				
	Agreement	Disagreement	Total	*κ*	*p*
	*n* (%)	*n* (%)			
Normal	22 (89)	3 (11)	25/66	0.89	0.000
Acute	6 (100)	0 (0)	6/66	0.69	0.001
Chronic	15 (89)	2 (11)	17/66	0.73	0.001
Acute plus chronic	16 (89)	2 (11)	18/66	0.62	0.005
Total	59 (89)	7 (11)	66/66	0.73	0.000
